# Role of Autologous Fat Grafting in the Conservative Treatment of Fecal Incontinence in Children

**DOI:** 10.3390/jcm12041258

**Published:** 2023-02-05

**Authors:** Valentina Pinto, Marco Pignatti, Giovanni Parente, Neil Di Salvo, Luca Contu, Mario Lima

**Affiliations:** 1 Division of Plastic Surgery, Department of Medical and Surgical Sciences for Children & Adults, University Hospital of Modena and Reggio Emilia, 41124 Modena, Italy; 2 Plastic Surgery Department, IRCCS Azienda Ospedaliero-Universitaria Sant’Orsola di Bologna, Via Massarenti 9, 40138 Bologna, Italy; 3 Dipartimento di Scienze Mediche e Chirurgiche (DIMEC), University of Bologna, Via Massarenti 9, 40138 Bologna, Italy; 4 Paediatric Surgery Department, IRCCS Azienda Ospedaliero-Universitaria Sant’Orsola di Bologna, Via Massarenti 9, 40138 Bologna, Italy

**Keywords:** anorectal malformations, fecal incontinence, pediatric fecal incontinence, autologous fat grafting, quality of life

## Abstract

Treatment of organic fecal incontinence in children, typical of anorectal malformations, is most often conservative; however, when necessary, it can be surgical. Autologous fat grafting, or lipofilling, can be used to improve fecal incontinence. We present our experience with the echo-assisted anal-lipofilling and its effects on fecal incontinence in children and on the quality of life of the entire family. Under general anesthesia, fat tissue was harvested according to the traditional technique, and processed in a closed system Lipogems^®^ set. Injection of the processed adipose tissue was guided by trans-anal ultrasound assistance. Ultrasound and manometry were also used for follow-up. From November 2018, we performed 12 anal-lipofilling procedures in six male patients (mean age 10.7 years). Five children had a stable improvement in bowel function with Krickenbeck’s scale scores going from soiling grade 3 pre-treatment in 100% of children to grade 1 post-treatment in 75% of them. No major post-operative complications developed. An increase in thickness of the sphincteric apparatus was shown at ultrasound during follow-up. The quality of life of the entire family, evaluated with a questionnaire, improved after the surgical treatment of the children. Anal-lipofilling is a safe and effective procedure to reduce organic fecal incontinence thereby benefiting both the patients and their families.

## 1. Introduction

Pediatric fecal incontinence, functional and organic, is a frequent problem estimated to affect 0.8–4.1% of children in Western societies. Anorectal malformations (ARMs), Hirschsprung disease, spinal defects, cerebral palsy, and myopathies are among its organic causes [1].

This condition greatly affects the patients’ social acceptance, especially during growth, and its daily management impacts on their parents’ social and familiar life [2].

Standard treatment of organic fecal incontinence is most often conservative; however, when necessary, it can be surgical. Traditional surgical procedures are usually complex and invasive, and children’s compliance is often low [3,4].

The medical instruments on which the physicians can count on have not improved in recent years and are essentially based on the use of regular enemas and osmotic laxatives. In patients not responding to standard treatment, the echo-assisted inter-sphincteric injection of autologous micro-fragmented adipose tissue (anal-lipofilling) can represent an effective option to control the loss of feces [5].

In the last 30 years, the use of autologous fat grafting has increased in plastic surgery and other surgical fields, being now considered both as a filler and a regenerative treatment.

The regenerative potential of fat grafting has been thoroughly investigated and it is attributed to the presence, in the grafted fat, of autologous adipose-derived stem cell (ADSC), that, through various mechanisms, improve soft tissue healing [6].

The injectable transplantation of small quantities of fat is a simple, effective, and reproducible technique that yields a high satisfaction rate and few complications. In children, adipose tissue is readily available and its harvesting is minimally invasive, requires a short procedure, and causes minor donor-site morbidity. Furthermore, the fat being autologous does not stimulate an immune response from the host. Because of these characteristics, autologous fat grafting is being increasingly applied also in pediatric malformative surgery.

Previous studies in adults [7,8,9] and our first study in a pediatric population [10] showed that, when used to treat fecal incontinence, the injected fat, in addition to its regenerative potential, acts as a bulky agent increasing the resting pressure, as verified with anorectal manometry, and thickens the anal sphincter, as documented by anal endo-sonography. 

Endoanal ultrasonography is a useful tool to guarantee a more precise injection of the fat in the inter-sphincteric plane [9].

We report here an extension of the preliminary results [10] of echo-assisted anal microfragmented lipofilling in a consecutive series of young patients, and the results of a survey that was administered to evaluate, in addition to the effectiveness of therapy in correcting fecal incontinence, the degree to which the quality of life (QoL) of the patients and their parents was modified.

## 2. Materials & Methods

We retrospectively included in the study, approved by the Ethical committee (CHPED-MAR-18-02), all children affected by fecal incontinence treated at our institution with autologous fat grafting from November 2018 to December 2020 who had a minimum follow-up of 9 months. In most children, fecal incontinence was ARM related, while in one child it was due to neurological impairment.

The patients and their parents were then invited to participate in a survey on quality of life. The Krickenbeck’s scale (KS) questionnaire was used to investigate the parents’ feeling over their child’s fecal incontinence before the procedure of anal-lipofilling and 6 months after it (Table 1).

In addition, the parents were asked to compare the quality of life of their family in the pre-treatment and the post-treatment periods (Table 2).

The patients (when appropriate) and their parents gave informed consent to participate in the research.

### 2.1. Anal-Lipofilling: Surgical Procedure 

The procedure was performed under general anesthesia and consisted of three distinct phases during a single surgical time: fat tissue harvesting, fat tissue processing, and injection of the processed adipose tissue.

#### 2.1.1. Fat Tissue Harvesting

Each child was examined preoperatively in the orthostatic position with the pinch test to precisely evaluate and define the area to be lipoaspirated. 

In children, the abdomen represents the ideal donor site because of fat availability and ease of access. In addition, the site is unique and median, and therefore, there is no need, to avoid morpho-volumetric asymmetries, to take bilateral samples as in the case of the hips, thighs, and buttocks. Further, when using the abdominal area as a donor site, the patient can be placed in the supine position, shortening the surgical time.

**Figure 1:**
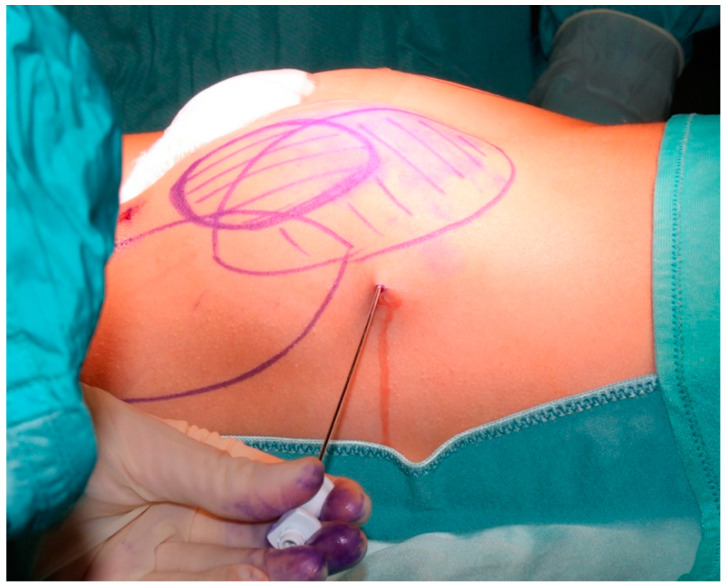
Harvesting of fat tissue in very thin patients (buttocks and supragluteal area).

In very thin children, the supra-gluteal region and inner knees are good alternative donor sites of adipose tissue (Figure 1); however, their use requires, as mentioned above, that bilateral sampling be performed to preserve the contouring of the buttocks, and that the child’s position at the time of the grafting be changed from prone to supine. The donor site is infiltrated with a solution of epinephrine 2 mcg/mL in saline (1:500), using an 18-Gauge cannula connected to a Luerlock^®^ syringe. After waiting 10 to 15 min to allow proper vasoconstriction, a liposuction cannula is introduced through a 2 mm incision.

The harvesting of an adequate amount of fat tissue (50–100 mL) is performed in a standardized fashion, as previously described, connecting a 20cc Vaclock^®^ syringe to a 3 mm 13-Gauge blunt cannula [7].

#### 2.1.2. Fat Tissue Processing

**Figure 2:**
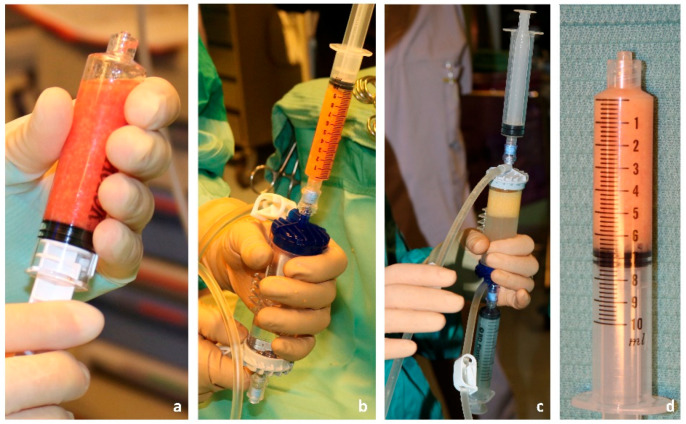
(**a**–**d**) Adipose tissue processing in the closed system.

Processing of the harvested adipose tissue was carried out in a closed system, using the Lipogems^®^ [11] set, a closed, full-immersion, low-pressure cylindrical system (Figure 2), to obtain a gradual volume reduction of the adipose tissue clusters and to remove impurities, producing an injectable fluid that contains a large number of pericytes and mesenchimal stem cells, the regenerative component of the subcutaneous tissue. Thanks to this system, fat tissue is micro-fragmented gently and proinflammatory oil and blood residues are washed away without the use of enzymes or other additives [12,13].

The purified fat graft was then transferred in smaller Luerlock^®^ syringes.

#### 2.1.3. Purified Tissue Injection 

**Figure 3:**
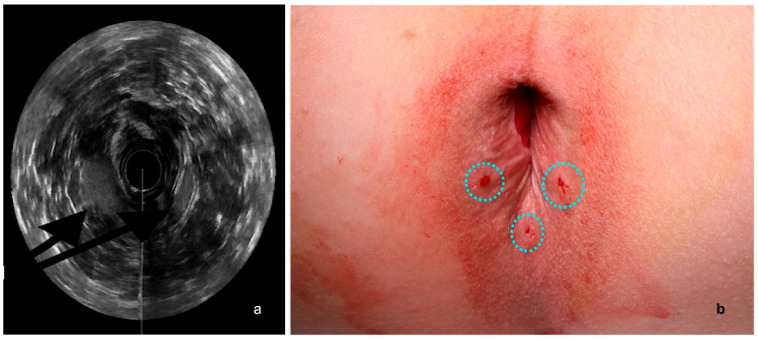
(**a**). Endosonographic axial sections of the middle anal canal after autologous fat grafting. The injected tissue is indicated by arrows. (**b**). Points of skin entrance of the cannula to reach the injection sites.

An ultrasound probe (anorectal 3D 2052 transducer—17 mm diameter, 13 MHz) was positioned endo-anally to provide images of the perianal tissues and to show the local anatomy in 3D. Guided by these live images, the surgeon can inject up to 10 mL of the processed fat in the inter-sphincteric plane in the anterior right, anterior left, and posterior points of the anal canal. (Figure 3a) The injection of the processed fat is performed in the described three sites (Figure 3b), avoiding the anterior point (12 o’clock) of the anal canal to protect the urethra from accidental damage. This choice, which minimizes the possible risks, derives from the traditional technique used before the ultrasound guidance became available.

### 2.2. US Assessment

Endoanal ultrasonography was performed in all the patients, before and during the procedure, and at 3 and 6 months of follow-up.

An anorectal 3D 2052 transducer (17 mm diameter, 13 MHz) with a BK-medical flexfocus 800 US system (BK Medical Italia SRL, Melegnano, 20077, Italy) was used for the echo assistance to evaluate the anatomy, to plan the injection site, and to visualize the changes in the thickness of the sphincteric apparatus.

With endoanal ultrasonography, the internal anal sphincter (IAS), the external anal sphincter (EAS), and the pubo-rectal sling were examined. In particular, the IAS thickness was registered as done in a regular endoanal ultrasonographic examination (IAS thickness is obtained as the average of measurements in three different quadrants of the sphincter, all taken at the same level of the sphincter). IAS thickness values were compared to the normal values for sex and age established by de la Portilla & Lopez-Alonso [14]; the mean IAS thickness values before and after the procedure were then compared. 

### 2.3. Manometry

To better understand the efficacy of anal lipofilling, we added the evaluation of the tone of the anal canal and continence ability using endoanal manometry before treatment and 3 months after fat grafting. 

A single pediatric surgeon performed all anorectal manometries with conventional ano-rectal manometry and a computerized system equipped with DYNO software 3000 (Medica s.p.a. Italy); a water perfused 4 radial holes silicone rubber catheter (outer diameter 4.5 mm) was used. The examination, not being painful, was performed without sedation to ensure patient cooperation. The operator measured the average resting pressure (ARP) and the average anal squeeze pressure, as an increment of ARP with maximum voluntary contraction in addition to the sphincter’s symmetry. 

### 2.4. Parents’ QoL Evaluation

Due to the young age of our patients, we felt the need to evaluate the quality of life and the social impact of the disorder on the entire family. To this end, we asked the parents of our patients to fill out a questionnaire divided into three sections (13 items) investigating Lifestyle (Scale 1), Depression/Self Perception (Scale 2), and Embarrassment (Scale 3), as shown in Table 2.

## 3. Results

From 2018, we performed 12 anal-lipofilling procedures in six male patients. The number of surgical procedures per patient was 2.0 ± 1.3 (range: 1–4), with a time interval between the procedures of 343.8 ± 220.1 days (range: 203–733 days) in children who underwent more than one surgery. The mean age was 10.7 ± 4.4 years (range: 6–17 years). Five children (83.3%) were affected by classical anorectal malformation: four children had recto-urethral fistulas and one patient had a recto-perineal fistula. In one child (*n* = 1; 16.7%) a tethered cord was at the basis of fecal incontinence. 

Five children (83,3%) had a stable improvement in bowel function at a median follow-up of 6 months, with improved KS scores going from soiling grade 3 pre-treatment in 100% of children to grade 1 post-treatment in 75% of them. 

Follow-up ranged from a minimum of 9 months to a maximum of 30 months

During the follow-up, the five patients who had benefited from the treatment maintained good control of anal continence. The child whose treatment was unsuccessful continues to be treated with enemas. 

No major post-operative complications were observed.

**Figure 4:**
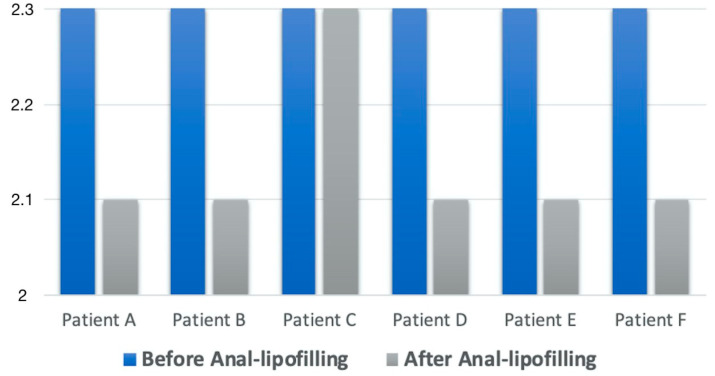
Results of anal lipofilling on soiling reported as the Krickenbeck scale (KS) score.

The characteristics of the patients and the surgical procedures are summarized in Table 3. Results of KS scores are reported in Figure 4.

### 3.1. US Assessment 

Endoanal ultrasonography was performed in all the patients, before and during the procedure, and at 3 and 6 months of follow-up.

Thus, in patients with ARM, pre-operative endoanal ultrasonography showed a thinner IAS compared to normal values for age and sex [12], while it was normal in the tethered cord patient

The pre-operative mean IAS thickness was 0.9 mm in ARM patients and 2.0 mm in the only patient with a tethered cord. 

Post-operative endoanal ultrasounds showed an increase in the IAS thickness in all patients. At 6 months of follow-up, the mean post-operative IAS thickness was 1.3 mm in the ARM patients and 2.2 mm in the tethered cord patient. The mean increase was 0.37 mm (33.6%). Detailed measurements are shown in Table 4.

### 3.2. Manometry 

We decided only after the start of the study to also evaluate the patients with ano-rectal manometry; therefore, at present, only two patients have undergone this test. 

One of the two was affected by fecal incontinence secondary to an anorectal malformation with recto-urethral fistula and had a thin and disrupted internal anal sphincter, as shown by US; the other suffered from neurological impairment due to a tethered cord. 

In the patient with the anorectal malformation, the manometric parameters were not modified by the surgical procedure. Accordingly, he did not obtain a clinical benefit from the procedure, nor an improvement at manometry. On the contrary, in the patient with the tethered cord, the ano-rectal manometry showed an increased resting pressure of the anal sphincter from 40 mmHg pre-operative to 60 mmHg after 6 months. No changes were evident in the anal squeeze pressure during maximum voluntary contraction. This finding corresponded clinically to improved control of the patient’s continence. 

The results of the evaluation of the parents’ QoL obtained with the described questionnaire (Table 2) are reported in detail in Table 5.

To summarize, the patients’ parents reported that limitations of family lifestyle (Scale 1), were “always or often present” in 74% of cases before treatment while they decreased to “sometimes or never present” in 91% of cases after treatment.

Both mothers and fathers reported depression and altered self-perception (Scale 2) “always or often” in 36.6% of cases before surgery, while it dropped to 3.33% after surgery.

Before surgery, embarrassment (Scale 3) was reported to be “often” present by 49.8% of parents, while after anal lipofilling was “sometimes” present in 58.1% 

## 4. Discussion 

Fecal incontinence is a significant clinical problem whose social impact can be classified according to the Krickenbeck criteria [15]. It is often due to anorectal malformations, which are the most prevalent congenital colorectal defects, occurring in approximately 1 to 3 in every 5000 live births [16]. Fecal incontinence in children should always initially be treated with dietary changes and accurate bowel management, powered, if necessary, by trans-anal irrigation. If such methods are ineffective, surgery should be considered [10].

The surgical correction of anorectal malformations can be either primary or secondary, i.e., after the creation of a colostomy. Currently, the most frequently adopted technique for all types of anorectal malformations is the posterior sagittal anorectoplasty (PSARP), although some rare forms require further surgical revision during growth [15]. 

Since the correction of fecal incontinence and anorectal malformations usually requires very invasive surgical techniques, other attempts with less aggressive surgeries have been attempted.

In particular, previous experiences in treating fecal incontinence in adults with various bulky agents [17] or with stem cell derived from human adipose tissue have been reported [7,8,9].

Hussain et al., in their systematic review [17], described various injectable agents, the techniques used for the treatment, and their safety and efficacy. The optimal injectable bulking agent should be biologically non-reactive, safe, nonbiodegradable, non-migratory, and easy to harvest and inject. Some bulking agents, such as collagen, silicone, synthetic non-particulate hydrogel, and Teflon, are often potentially associated with significant local and systemic adverse effects and with the risk of infection [18,19].

A randomized controlled trial [20] reported on 40 patients (mean age 59.5 years vs. 58.9 years) randomized to have inter-sphincteric injection of silicone biomaterial or submucosal injection of carbon-coated beads (Durasphere). In addition to being studied with anorectal physiology and endoanal ultrasound, they were investigated with a validated incontinence score and quality of life questionnaires. In patients with internal sphincter dysfunction, injectable silicone biomaterial was safer and more effective than carbon-coated beads. In addition, the patients in the silicone group reported a significant improvement in the fecal incontinence quality of life scale. 

A Cochrane Database Systemic Review [21] identified five eligible randomized trials with a total of 382 patients. Most trials reported a short-term benefit from injections regardless of the material used, including placebo saline injection. Dextranomer in stabilized hyaluronic acid seems to be more effective than placebo, but with more adverse effects. The authors concluded that the use of this substance as a bulking agent improves, in the short term, continence, for slightly more than half of the patients. However, the number of identified trials was limited and none of them reported patient evaluation of outcomes. 

Quiroz and colleagues also showed in a very recent study [22] the safety and efficacy of Non-animal Stabilized Hyaluronic Acid/Dextranomer.

Hong et al. [23] reviewed the midterm outcomes of 11 different injectable bulking agents in 889 patients reported in 23 articles. The weighted mean follow-up duration was 23.7 months. The procedures resulted in significant midterm improvement. Implants injected through a perianal route were intact at ultrasound, which provided higher improvement in incontinence.

The use of adipose tissue for fecal incontinence in adults goes back to the 1990’s when Shafik first reported his experience [7] with autologous fat grafting in 14 patients; he described noticeable fat reabsorption, and the need for multiple sessions. 

Bernardi, in 1998 [8], described a single successful case of an adult patient treated with two sessions of autologous fat grafting. 

More recently, Sarveazad et al. [9] performed a randomized double-blind clinical trial on patients with sphincter defects (nine patients and nine controls) to investigate the ability of stem cells derived from human adipose tissue to improve anal sphincter incontinence after sphincteroplasty.

Using electromyography and endorectal sonography, they found that the ratio of the area occupied by the muscle to the total area of the lesion was 7.91% greater in the cell group compared with the control group. The conclusion of the study was that the injection of adipose-derived stem cells during repair surgery for fecal incontinence might replace the fibrous tissue with contractile muscle tissue.

The hypothesized mechanism of action is that the injectable or implantable agents embedded in the anal canal improve sphincter contractility by increasing the sarcomere fiber length of the canal, in addition to providing a bulking effect. 

Sarveazad et al. [9] showed an increase in the muscle cells and proposed that this effect was due to the high levels of growth factors secreted by the adipose-derived stem cells that have the potential to differentiate into muscle cells.

Concerning the pediatric population, autologous fat grafting, thanks to its low invasiveness and low rate of complications, has been used for the treatment of selected disorders, proving it to be a safe and effective technique [24,25,26].

Autologous fat, also called lipofilling, due to its regenerative potential, can be considered more than just a filler. In fact, it provides progenitor cells that, under the effect of the local environment, differentiate into cell lines useful to reduce the local damage and repair or mitigate the loss of function [27].

Consequently, the technique has become a new precious tool for the treatment of malformative or degenerative conditions. 

Lipofilling is a surgical technique that collects, through a liposuction procedure, fat from the anatomical areas where it is naturally abundant, such as thighs, flanks, knees, and abdomen. After harvesting, the material is processed either by decantation, filtration, or centrifugation.

Processing allows us to separate and discard the fluid that was infiltrated for the liposuction, and the oil derived from the damage of adipocytes, while the rest is re-injected into the patient’s areas needing treatment. The injection technique must be accurate because only small amounts of the tissue grafted into vital tissues will be perfused and survive [28].

Lipofilling has been used since the 1890s, with questionable results, to transfer whole portions of subcutaneous tissue. Starting in the 1920s and, more effectively, in recent decades, the fat is being transferred in small quantities of fragmented tissue.

A radical change in regenerative surgery happened in the 1990s when plastic surgeons noticed some unexpected beneficial effects of autologous fat grafting on damaged or fibrotic tissues [29].

In the years following the first experiences by Coleman, Rigotti, and Kouri [30,31,32], several studies provided a deeper knowledge of the procedure and into the cellular events involved.

In particular, it was discovered that the beneficial effects on the tissues were due to the presence of the so-called Adipose Derived Stem Cells, residing into the stromal vascular fraction of the transplanted fat, a niche located at the junction between the vessels nourishing the fat and the adipocytes. Progenitor cells of the lines of pericites, osteoblasts, adipocytes, and fibroblasts contained in the fat were able to differentiate and grow according to the environment where they were transplanted, with a regenerative effect on the surrounding tissues [6,33,34]. 

Several pediatric conditions, such as congenital craniofacial malformations and post-traumatic/iatrogenic or post-oncological facial asymmetries, are now often treated with autologous fat grafting [26]. More recently, autologous fat grafting has been successfully used also to regenerate atrophic or fibrotic skin due to a large number of clinical conditions [35], such as radiodermatitis, burning scars, systemic autoimmune connective tissue diseases including scleroderma, and different types of morphea [36]. The regenerative potential of fat grafting has also been used to treat functional disorders such as velopharyngeal incompetence [37,38] or vocal folds paralysis [39].

An evolution of the classical Coleman lipofilling technique was aimed to decrease the size of the clusters of lipoaspirate to improve their engraftment. 

The microfragmentation technology (Lipogems^®^) improves and optimizes the natural properties of the adipose tissue, without the use of enzymes, additives, or separation centrifugation. The entire procedure is performed in a single surgical time and can be repeated without compromising, if necessary, subsequent more invasive surgical procedures. 

Microfragmented autologous fat amount contains a stem cell population with clearly distinctive traits when compared to the classical lipoaspirate. Properties of the microfragmented fat injected have been extensively studied and characterized in vitro by other authors [40,41].

The use of microfragmented autologous fat grafting in our patients improved their continence, as shown by the Krickenbeck scale (KS) score and, as a consequence, the quality of life of their families. Our preliminary results, although in a small population, seem promising. Evaluation in a larger series with a controlled study and a longer follow-up will be needed. The major disadvantage of the technique is due to the unpredictable quota of fat resorption and, therefore, the need for multiple sessions.

KS has become the gold standard for the classification of anorectal malformations. This scale is an internationally validated useful and simple way to determine the type of anorectal malformation and the functional outcomes after surgery. It also compares different surgical procedures, defining standards for pre-operative and post-operative management, allowing different centers to compare clinical data [42].

Endoanal ultrasound increases the accuracy of location and amount of fat injection. 

During follow-up, the five patients who had benefited from the treatment maintained good control of anal continence. Longer follow-up studies need to be performed to confirm the duration of the positive effect. We believe that the observed efficacy was a consequence of the regenerative potential of the transplanted fat tissue. 

The attention to the family problems caused by the clinical conditions of the affected child is a crucial part of the management of anorectal malformations-related fecal incontinence.

The health problems caused by anorectal malformations interfere not only with a child’s psychological development and social interactions, but could also disrupt the family’s life and cohesion. Therefore, at present, the optimal approach to fecal incontinence due to anorectal malformations or other defects, not responding to medical measures, includes the autologous fat grafting-regenerative approach in addition to psychological support to the patient and his/her family.

## 5. Conclusions

The treatment of fecal incontinence consists of a wide range of surgical and non-surgical procedures. Fat grafting is a simple, effective, and reproducible technique, with a high satisfaction rate and few complications. When non-surgical techniques are not sufficient, fat grafting, with its moderately invasive technique, may be attempted before more aggressive surgeries. According to our preliminary experience, fat tissue represents an adequate therapeutic option for the majority of patients with anorectal malformations, thanks to the bulking effect and the regenerative potential of the adipose derived stem cells, without compromising further treatments.

## Figures and Tables

**Table 1:** International classification (Krickenbeck’s scale). KS represents the gold standard for the classification of AnoRectal Malformations, divided in three sections (1. Voluntary bowel movements, 2. Soiling/fecal incontinence, 3. Constipation), used in the management of the treatment.

1. Voluntary Bowel Movements
Feeling of urge, capacity to verbalize and hold the bowel movements 1.0. No. 1.1. Yes.
2. Soiling/fecal incontinence
2.1. No. 2.2. Occasionally (once/twice a week). 2.3. Every day, no social problem. 2.4. Constant, social problem.
3. Constipation
3.1. No. 3.2. Manageable with diet. 3.3. Requires laxatives. 3.4. Resistant to diet and laxatives.

**Table 2:** Parents’ QoL questionnaire.

	Questions	Answers					
		1	2	3	4	5	6
**A**	**Scale 1: Lifestyle**						
**1**	I cannot do many of things I want to do with my family and with other families	❑ Always ❑ Sometimes ❑ Often ❑ Never					
**2**	I am afraid to go out like going to a movie or to church						
**3**	I avoid traveling by plane or train						
**4**	I avoid visiting friends						
**5**	I avoid going out to eat						
**6**	I avoid staying overnight away from home with friends and their families						
**B**	**Scale 2: Depression/Self Perception**						
**1**	I feel different from other mothers/parents	❑ Always ❑ Sometimes ❑ Often ❑ Never					
**2**	I enjoy my family less						
**3**	I feel depressed						
**4**	The relationship with my husband and other children suffers						
**5**	I’m worried that my son is distracted at school during lessons and miss school days for visits and operations						
**C**	**Scale 3: Embarrassment**						
**1**	I worry about my son leaks stool without even knowing it and others smelling stool on him/her I worry children make fun of him/her	❑ Always ❑ Sometimes ❑ Often ❑ Never					
**2**	It makes me depressed to think that teachers or other mothers might believe that I did not wash my child properly or I did not educate him						

**Table 3:** Patient and surgery characteristics.

***Data Collection***		
**DEMOGRAPHIC AND ** **CLINICAL DATA**	**N° of patients treated**	6
	**Gender**	100% M
	**Age at surgery**	10.7 ± 4.4 years (range: 6–17 years)
	**Causes ** **of fecal incontinence**	anorectal malformation 83% (5) n = 4 recto-urethral fistulas n = 1 recto-perineal fistula tethered cord 17% (1)
**CLINICAL ** **ASSESSMENT**	**Preoperative KS score**	100% (6): 1.0, 2.3, 3.0
	**Postoperative KS score**	83% (5): 1.0, 2.1, 3.0 17% (1): 1.0, 2.3, 3.0
**SURGICAL ** **DATA**	**N° of procedures**	12
	**N° of procedures per patients**	2.0 ± 1.3 (range: 1–4)
	**Interval time between the procedures**	343.8 days ± 220.1 days (range 203–733 days)
	**Pre-op IAS thickness ** **(mean)**	0.9 mm in ARM patients (5); 2.0 mm tethered cord patient (1)
	**Post-op IAS thickness ** **(mean)**	1.3 mm in ARM patients (5); 2.2 mm tethered cord patient (1)

**Table 4:** Internal anal sphincter thickness (mm) measured with ultrasound pre-operatively and 6 months after the last treatment with echo-assisted lipofilling in our six incontinent patients.

Patient	Pre-Existing Condition	Pre-Operative AS Thickness (mm)	Post-Operative IAS Thickness (mm)
1.	ARM with recto-urethral fistula	0.6	1.00
2.	ARM with recto-urethral fistula	1.0	1.2
3.	ARM with recto-urethral fistula	0.9	1.4
4.	ARM with recto-urethral fistula	0.8	1.2
5.	ARM with recto-perineal fistula	1.1	1.5
6.	Normal ano-rectal anatomy and tethered cord	2.0	2.2

**Table 5:** The results of the evaluation of the parents’ quality of life obtained with the described questionnaire (Table 2) are reported in detail.

Questions	Pre-Surgical Answers				Post-Surgical Answers			
	Always	Often	Sometimes	Never	Always	Often	Sometimes	Never
**A—Scale 1: Lifestyle**								
**A1**	0% (*N =* 0)	100% (*N =* 6)	0% (*N =* 0)	0% (*N =* 0)	0% (*N =* 0)	16.7% (*N =* 1)	83.3% (*N =* 5)	0% (*N =* 0)
**A2**	16.7% (*N =* 1)	83.3% (*N =* 5)	0% (*N =* 0)	0% (*N =* 0)	0% (*N =* 0)	16.7% (*N =* 1)	83.3% (*N =* 5)	0% (*N =* 0)
**A3**	16.7% (*N =* 1)	83.3% (*N =* 5)	0% (*N =* 0)	0% (*N =* 0)	0% (*N =* 0)	16.7% (*N =* 1)	83.3% (*N =* 5)	0% (*N =* 0)
**A4**	16.7% (*N =* 1)	33.3% (*N =* 2)	33.3% (*N =* 2)	16.7% (*N =* 1)	0% (*N =* 0)	0% (*N =* 0)	83.3% (*N =* 5)	16.7% (*N =* 1)
**A5**	16.7% (*N =* 1)	33.3% (*N =* 2)	33.3% (*N =* 2)	16.7% (*N =* 1)	0% (*N =* 0)	0% (*N =* 0)	83.3% (*N =* 5)	16.7% (*N =* 1)
**A6**	16.7% (*N =* 1)	33.3% (*N =* 2)	33.3% (*N =* 2)	16.7% (*N =* 1)	0% (*N =* 0)	0% (*N =* 0)	83.3% (*N =* 5)	16.7% (*N =* 1)
**AGGREGATED A**	**13.8% (*N =* 5)**	**60.1% (*N =* 22)**	**16.6% (*N =* 6)**	**8.31 (*N =* 3)**	**0% ** **(*N =* 0)**	**8.31% (*N =* 3)**	**83.1% (*N =* 30)**	**8.31% (*N =* 3)**
**B—Scale 2: Depression/ ** **Self-Perception**								
**B1**	0% (*N =* 0)	16.7% (*N =* 1)	33.3% (*N =* 2)	50% (*N =* 3)	0% (*N =* 0)	0% (*N =* 0)	33.3% (*N =* 2)	66.7% (*N =* 4)
**B2**	16.7% (*N =* 1)	33.3% (*N =* 2)	16.7% (*N =* 1)	33.3% (*N =* 2)	0% (*N =* 0)	0% (*N =* 0)	66.7% (*N =* 4)	33.3% (*N =* 2)
**B3**	33.3% (*N =* 2)	33.3% (*N =* 2)	16.7% (*N =* 1)	16.7% (*N =* 1)	16.7% (*N =* 1)	0% (*N =* 0)	66.7% (*N =* 4)	16.7% (*N =* 1)
**B4**	0% (*N =* 0)	16.7% (*N =* 1)	50% (*N =* 3)	33.3% (*N =* 2)	0% (*N =* 0)	0% (*N =* 0)	66.7% (*N =* 4)	33.3% (*N =* 2)
**B5**	16.7% (*N =* 1)	16.7% (*N =* 1)	66.7% (*N =* 4)	0% (*N =* 0)	0% (*N =* 0)	0% (*N =* 0)	100% (*N =* 6)	0% (*N =* 0)
**AGGREGATED B**	**13.3% (*N =* 4)**	**23.3% (*N =* 7)**	**36.6% (*N =* 11)**	**26.6% ** **(*N =* 8)**	**3.33% (*N =* 1)**	**0% (*N =* 0)**	**66.6% (*N =* 20)**	**29.97% (*N =* 9)**
**C—Scale 3: Embarrassment**								
**C1**	0% (*N =* 0)	83.3% (*N =* 5)	16.7% (*N =* 1)	0% (*N =* 0)	0% (*N =* 0)	16.7% (*N =* 1)	83.3% (*N =* 5)	0% (*N =* 0)
**C2**	0% (*N =* 0)	16.7% (*N =* 1)	16.7% (*N =* 1)	66.7% (*N =* 4)	0% (*N =* 0)	0% (*N =* 0)	33.3% (*N =* 2)	66.7% (*N =* 4)
**AGGREGATED C**	**0% (*N =* 0)**	**49.8% (*N =* 6)**	**16.6% (*N =* 2)**	**33.2% (*N =* 4)**	**0% (*N =* 0)**	**8.3% (*N =* 1)**	**58.1% (*N =* 7)**	**33.2% ** **(*N =* 4)**

## Data Availability

Data is unavailable due to privacy and ethical restrictions.

## Abbreviations

ADSC	adipose-derived stem cell
ARMs	Ano-Rectal Malformations
ARP	average resting pressure
ASP	anal squeeze pressure
KS	Krickenbeck scale
MCV	maximum voluntary contraction
PSARP	posterior sagittal anorectoplasty
QoL	quality of life
SVF	stromal vascular fraction

## References

[1] Rajindrajith S., Devanarayana N.M., Benninga M.A. (2013). Review article: Faecal incontinence in children: Epidemiology, pathophysiology, clinical evaluation and management. Aliment. Pharmacol. Ther..

[2] Kyrklund K., Neuvonen M.I., Pakarinen M.P., Rintala R.J. (2018). Social Morbidity in Relation to Bowel Functional Outcomes and Quality of Life in Anorectal Malformations and Hirschsprung’s Disease. Eur. J. Pediatr. Surg..

[3] Molina M.E., Lema A., Palacios M.G., Somoza I., Gómez J.V., Tellado M.G., Pais E., Dargallo T., Vela D. (2010). Quality of life in children operated on for anal atresia. Cir. Pediatr..

[4] Grasshoff-Derr S., Backhaus K., Hubert D., Meyer T. (2011). A successful treatment strategy in infants and adolescents with anorectal malformation and incontinence with combined hydrocolonic ultrasound and bowel management. Pediatr. Surg. Int..

[5] Jeong H., Hwang S.H., Kim H.R., Ryu K.O., Lim J., Yu H.M., Yoon J., Kim C.Y., Jeong K.Y., Jung Y.J. (2019). Effectiveness of Autologous Fat Graft in Treating Fecal Incontinence. Ann. Coloproctol..

[6] Naderi N., Combellack E.J., Griffin M., Sedaghati T., Javed M., Findlay M.W., Wallace C.G., Mosahebi A., Butler P.E., Seifalian A.M. (2017). The regenerative role of adipose-derived stem cells (ADSC) in plastic and reconstructive surgery. Int. Wound J..

[7] Shafik A. (1995). Perianal injection of autologous fat for treatment of sphincteric incontinence. Dis. Colon. Rectum..

[8] Bernardi C., Favetta U., Pescatori M. (1998). Autologous fat injection for treatment of fecal incontinence: Manometric and echographic assessment. Plast. Reconstr. Surg..

[9] Sarveazad A., Newstead G.L., Mirzaei R., Joghataei M.T., Bakhtiari M., Babahajian A., Mahjoubi B. (2017). A new method for treating fecal incontinence by implanting stem cells derived from human adipose tissue: Preliminary findings of a randomized double-blind clinical trial. Stem Cell Res. Ther..

[10] Parente G., Pinto V., Di Salvo N., D’Antonio S., Libri M., Gargano T., Catania V.D., Ruggeri G., Lima M. (2020). Preliminary Study on the Echo-Assisted Intersphincteric Autologous Microfragmented Adipose Tissue Injection to Control Fecal Incontinence in Children Operated for Anorectal Malformations. Children.

[11] Tremolada C., Colombo V., Ventura C. (2016). Adipose Tissue and Mesenchymal Stem Cells: State of the Art and Lipogems® Technology Development. Curr. Stem Cell Rep..

[12] Bianchi F., Maioli M., Leonardi E., Olivi E., Pasquinelli G., Valente S., Mendez A.J., Ricordi C., Raffaini M., Tremolada C. (2013). A new nonenzymatic method and device to obtain a fat tissue derivative highly enriched in pericyte-like elements by mild mechanical forces from human lipoaspirates. Cell Transplant..

[13] Ceserani V., Ferri A., Berenzi A., Benetti A., Ciusani E., Pascucci L., Bazzucchi C., Coccè V., Bonomi A., Pessina A. (2016). Angiogenic and anti-inflammatory properties of micro-fragmented fat tissue and its derived mesenchymal stromal cells. Vasc. Cell..

[14] De la Portilla F., López-Alonso M. (2009). Endosonography of the anal canal: Findings in children. Dis. Colon. Rectum..

[15] Holschneider A., Hutson J., Peña A., Beket E., Chatterjee S., Coran A., Davies M., Georgeson K., Grosfeld J., Gupta D. (2005). Preliminary report on the International Conference for the Development of Standards for the Treatment of Anorectal Malformations. J. Pediatr. Surg..

[16] Van den Hondel D., Sloots C.E., Gischler S.J., Meeussen C.J., Wijnen R.M., IJsselstijn H. (2013). Prospective long-term follow up of children with anorectal malformation: Growth and development until 5 years of age. J. Pediatr. Surg..

[17] Hussain Z.I., Lim M., Stojkovic S.G. (2011). Systematic review of perianal implants in the treatment of faecal incontinence. Br. J. Surg..

[18] Vaizey C.J., Kamm M.A. (2005). Injectable bulking agents for treating fecal incontinence. Br. J. Surg..

[19] Malizia A.A., Rushton H.G., Woodard J.R., Newton N.E., Reiman H.M., Lopez O.F. (1987). Migration and granulomatous reaction after intravesical subureteric injection of polytef. J. Urol..

[20] Tjandra J.J., Chan M.K., Yeh H.C. (2009). Injectable silicone biomaterial (PTQ) is more effective than carbon-coated beads (Durasphere) in treating passive faecal incontinence--a randomized trial. Color. Dis..

[21] Maeda Y., Laurberg S., Norton C. (2013). Perianal injectable bulking agents as treatment for faecal incontinence in adults. Cochrane Database Syst. Rev..

[22] Quiroz L.H., Galliano DEJr da Silva G., Carmichael J.C., Pan L.C., Bromley E.R., Hinahara J., Goss T.F. (2022). Efficacy and Safety of a Non-animal Stabilized Hyaluronic Acid/Dextranomer in Improving Fecal Incontinence: A Prospective, Single-Arm, Multicenter, Clinical Study with 36-Month Follow-up. Dis. Colon. Rectum..

[23] Hong K.D., Kim J.S., Ji W.B., Um J.W. (2017). Midterm outcomes of injectable bulking agents for fecal incontinence: A systematic review and meta-analysis. Technol. Coloproctol..

[24] Cantarella G., Mazzola R.F., Mantovani M., Mazzola I.C., Baracca G., Pignataro L. (2012). Fat injections for the treatment of velopharyngeal insufficiency. J. Craniofac. Surg..

[25] Koonce S.L., Grant D.G., Cook J., Stelnicki E.J. (2018). Autologous Fat Grafting in the Treatment of Cleft Lip Volume Asymmetry. Ann. Plast. Surg..

[26] Shih L., Abu-Ghname A., Davis M.J., Xue A.S., Dempsey R.F., Buchanan E.P. (2020). Applications of Fat Grafting in Pediatric Patients. Semin. Plast. Surg..

[27] Krastev T.K., Schop S.J., Hommes J., Piatkowski A., van der Hulst R.R.W.J. (2020). Autologous fat transfer to treat fibrosis and scar-related conditions: A systematic review and meta-analysis. J. Plast. Reconstr. Aesthet. Surg..

[28] Coleman S.R. (2001). Structural fat grafts: The ideal filler?. Clin. Plast. Surg..

[29] Coleman S.R. (2002). Hand rejuvenation with structural fat grafting. Plast. Reconstr. Surg..

[30] Coleman S.R. (1995). Long-term survival of fat transplants: Controlled demonstrations. Aesthetic Plast. Surg..

[31] Galiè M., Pignatti M., Scambi I., Sbarbati A., Rigotti G. (2008). Comparison of different centrifugation protocols for the best yield of adipose-derived stromal cells from lipoaspirates. Plast. Reconstr. Surg..

[32] Kosowski T.R., Rigotti G., Khouri R.K. (2015). Tissue-Engineered Autologous Breast Regeneration with Brava®-Assisted Fat Grafting. Clin. Plast. Surg..

[33] Piccinno M.S., Veronesi E., Loschi P., Pignatti M., Murgia A., Grisendi G., Castelli I., Bernabei D., Candini O., Conte P. (2013). Adipose stromal/stem cells assist fat transplantation reducing necrosis and increasing graft performance. Apoptosis.

[34] Rehman J., Traktuev D., Li J., Merfeld-Clauss S., Temm-Grove C.J., Bovenkerk J.E., Pell C.L., Johnstone B.H., Considine R.V., March K.L. (2004). Secretion of angiogenic and antiapoptotic factors by human adipose stromal cells. Circulation.

[35] Coleman S.R. (2006). Structural fat grafting: More than a permanent filler. Plast. Reconstr. Surg..

[36] Pignatti M., Spinella A., Cocchiara E., Boscaini G., Lusetti I.L., Citriniti G., Lumetti F., Setti G., Dominici M., Salvarani C. (2020). Autologous Fat Grafting for the Oral and Digital Complications of Systemic Sclerosis: Results of a Prospective Study. Aesthetic Plast Surg..

[37] Mazzola R.F., Cantarella G., Torretta S., Sbarbati A., Lazzari L., Pignataro L. (2011). Autologous fat injection to face and neck: From soft tissue augmentation to regenerative medicine. Acta Otorhinolaryngol. Ital..

[38] Mazzola R.F., Cantarella G., Mazzola I.C. (2015). Regenerative Approach to Velopharyngeal Incompetence with Fat Grafting. Clin. Plast. Surg..

[39] Lahav Y., Malka-Yosef L., Shapira-Galitz Y., Cohen O., Halperin D., Shoffel-Havakuk H. (2021). Vocal Fold Fat Augmentation for Atrophy, Scarring, and Unilateral Paralysis: Long-term Functional Outcomes. Otolaryngol. Head Neck Surg..

[40] Vezzani B., Shaw I., Lesme H., Yong L., Khan N., Tremolada C., Péault B. (2018). Higher Pericyte Content and Secretory Activity of Microfragmented Human Adipose Tissue Compared to Enzymatically Derived Stromal Vascular Fraction. Stem Cells Trans. Med..

[41] Laureti S., Gionchetti P., Cappelli A., Vittori L., Contedini F., Rizzello F., Golfieri R., Campieri M., Poggioli G. (2020). Refractory Complex Crohn’s Perianal Fistulas: A Role for Autologous Microfragmented Adipose Tissue Injection. Inflamm. Bowel Dis..

[42] Van der Steeg H.J., Schmiedeke E., Bagolan P., Broens P., Demirogullari B., Garcia-Vazquez A., Grasshoff-Derr S., Lacher M., Leva E., Makedonsky I. (2015). European consensus meeting of ARM-Net members concerning diagnosis and early management of newborns with anorectal malformations. Technol. Coloproctol..

